# The Performance of ChatGPT-4V in Interpreting Images and Tables in the Japanese Medical Licensing Exam

**DOI:** 10.2196/54283

**Published:** 2024-05-23

**Authors:** Soshi Takagi, Masahide Koda, Takashi Watari

**Affiliations:** 1 Faculty of Medicine, Shimane University, Izumo, Japan; 2 Co-learning Community Healthcare Re-innovation Office, Graduate School of Medicine, Dentistry and Pharmaceutical Sciences, Okayama University, Okayama, Japan; 3 General Medicine Center, Shimane University Hospital, Izumo, Japan; 4 Integrated Clinical Education Center, Kyoto University Hospital, Kyoto, Japan

**Keywords:** ChatGPT, medical licensing examination, generative artificial intelligence, medical education, large language model, images, tables, artificial intelligence, AI, Japanese, reliability, medical application, medical applications, diagnostic, diagnostics, online data, web-based data

## Introduction

OpenAI’s ChatGPT, a leading large language model (LLM), has shown promise for medical purposes. The program can pass the United States Medical Licensing Examination (USMLE) and the Japanese Medical Licensing Exam (JMLE) [1-3]. However, previous studies regarding this software have focused on its text-based capabilities. ChatGPT-4 Vision (ChatGPT-4V), announced on September 25, 2023, includes image input features, potentially expanding the medical applications of the program [4]. To assess the multimodal performance of ChatGPT-4V in medicine, its performance on JMLE questions involving clinical images and tables was tested.

## Methods

### Overview

ChatGPT-4V was used to complete the 117th JMLE in the Japanese language (Figure S1 in Multimedia Appendix 1). Its responses were compared to the passing criteria and mean human examinee score of the JMLE. This study, conducted from October 12 to 14, 2023, used the September 25, 2023, version of the LLM (ChatGPT-4V) with a knowledge cutoff date of January 2022 (Multimedia Appendix 2 [5]). Human examinees’ correct response rates were obtained from statistics based on reports from actual JMLE examinees, calculated by medu4, a preparatory school for the JMLE [5,6].

### Statistical Analysis

The mean and 95% CIs of the test scores are provided. A one-sample proportion test was used to compare the correct response rate of the human examinees with that of ChatGPT-4V. Statistical significance was set at *P*<.05 for all 2-tailed tests. All statistical analyses were conducted using Stata statistical software (version 17; StataCor).

### Ethical Considerations

This study used previously available web-based data and did not include human participants. Therefore, Shimane University’s Institutional Review Board did not mandate ethics approval.

## Results

### Evaluation Outcomes

The responses to 386 questions from the 117th JMLE were used in this study. Using the Ministry of Health, Labor, and Welfare criteria, GPT-4V scored 85.1% on the essential knowledge section and 76.5% on the other sections of the JMLE, meeting the passing criteria [6]. For text-only questions, ChatGPT-4V achieved a correct response rate of 84.5%, similar to the mean human examinee score (Table 1). The correct response rate for questions with images was 71.9% for ChatGPT-4V, 13.1 points below the mean human examinee score (*P*<.001). The correct response rate for questions with tables (including figures) was 35.0% for ChatGPT-4V, which was significantly lower than the mean human examinee score (83.9%; *P*<.001).

**Table 1.** Correct response rates of ChatGPT-4 Vision (ChatGPT-4V) and human examinees on the Japanese Medical Licensing Examination (JMLE).

Characteristics		Total, n (%)		Examinees^a^, mean	GPT-4V, mean	95% CI	Difference	*P* value
All questions		386 (100)		84.9	78.2	74.1-82.4	−6.7	.003
**Question category**								
	Essential knowledge	96 (24.9)		89.6	83.3	75.9-90.8	−6.3	.04
	General clinical knowledge	144 (37.3)		83.1	70.8	63.4-78.3	−12.3	<.001
	Specific diseases	146 (37.8)		83.5	82.2	76.0-88.4	−1.3	.67
**Type**								
	General	190 (49.2)		84.6	78.9	73.2-84.7	−5.7	.03
	Clinical	149 (38.6)		84.1	77.2	70.4-83.0	−6.9	.02
	Clinical sentence	47 (12.2)		88.5	78.7	67.0-90.4	−9.8	.04
**Imaging and table questions**								
	Text only	252 (65.3)		84.9	84.5	80.1-89.0	−0.4	.87
	With images	114 (29.5)		85.0	71.9	63.7-80.2	−13.1	<.001
	With tables	20 (5.2)		83.9	35.0	14.1-55.9	−48.9	<.001

a The correct response rates of human examinees are based on a survey of actual human examinees, reported by medu4, a preparatory school for the JMLE [5].

## Discussion

### Principal Results

Although ChatGPT-4V demonstrated proficiency in text-centric questions, the correct response rates were significantly lower for image and table-oriented questions. ChatGPT-4V may have poorer text comprehension skills compared to ChatGPT-4, even when image processing is not required [7]. Additionally, a language bias may obscure the image context when interpreting images and texts simultaneously, potentially leading to an overreliance on prior text information, even when it contradicts the image context, a phenomenon called “hallucination” [8]. These factors may have led to ChatGPT-4V’s lower rate of correct responses to questions involving images.

Furthermore, responding to questions with tables requires interpreting the Japanese characters within the tables. OpenAI has verified that its GPT-4V model misrecognizes symbols, including image characters [4]. Previous studies have noted that GPT-4V relies on text-based information rather than an analysis of tables when answering questions [8]. In addition, the program’s performance diminishes when interpreting characters in non-Latin languages [9]. These factors may explain the observed decline in performance when interpreting tables containing Japanese characters.

The multimodal LLM GPT-4V is unreliable in interpreting information presented in image or tables, especially for medical purposes [4]. Further development of the program is required for diagnostic applications.

### Limitations

This study has several limitations. First, different results may be obtained even when using the same methods owing to the inherent randomness of ChatGPT or version changes in ChatGPT. A report indicates that test results can vary with repeated responses from ChatGPT [10]. Furthermore, when providing images to ChatGPT, we did not remove blank spaces, indicating that the quality of images sent to ChatGPT could also affect the outcomes. Second, the JMLE includes options that, if selected twice or more, will result in failure. However, these options are not publicly disclosed, making them unaccounted for in this study [5]. Finally, although this study focused on ChatGPT, ongoing advancements in other multimodal LLMsshould also be considered.

### Conclusions

ChatGPT-4V successfully passed the 117th JMLE, demonstrating proficiency in handling including image- and table-based questions. However, more developments are needed to improve its ability to interpret tables. Further research should assess the safety and efficacy of ChatGPT-4V as a multimodal LLM in supporting medical practice, facilitating learning in clinical environments and advancing medical education.

## Supplementary material

10.2196/54283 Multimedia Appendix 1 Additional statistics.

10.2196/54283 Multimedia Appendix 2 Detailed methodology.

## Abbreviations

ChatGPT-4V: ChatGPT 4 Vision
JMLE: Japanese Medical Licensing Examination
LLM: large language model
USMLE: United States Medical Licensing Examination
