# Supplementary material for: The Performance of ChatGPT-4V in Interpreting Images and Tables in the Japanese Medical Licensing Exam
Source: JMIR Med Educ. 2024 May 23;10:e54283. doi: 10.2196/54283 (PMC11148840; doi:10.2196/54283)

Supplementary File 1

Figure S1. Screenshot of an actual ChatGPT-4V(ion) response: text-only questions


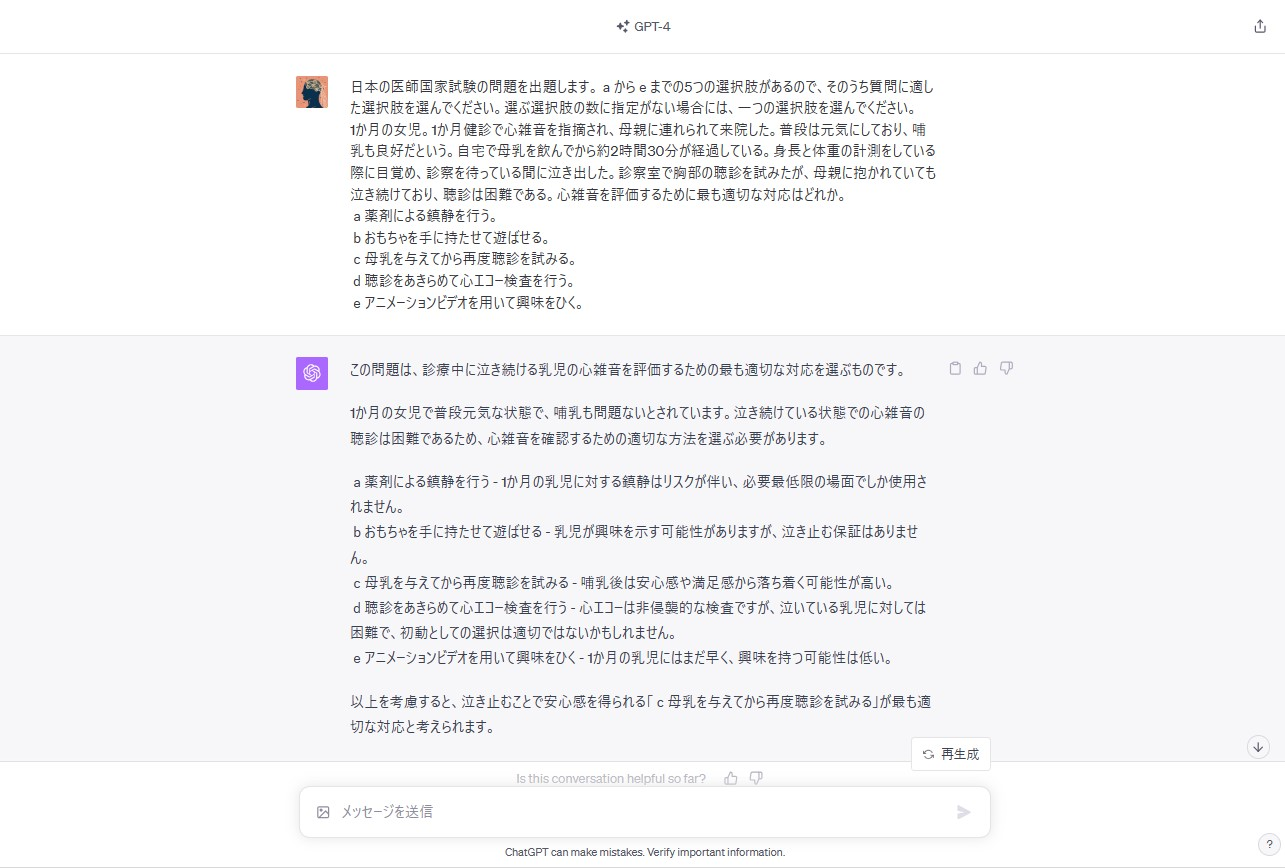


Figure S2. Screenshot of an actual ChatGPT-4V(ion) response: question with X-ray image


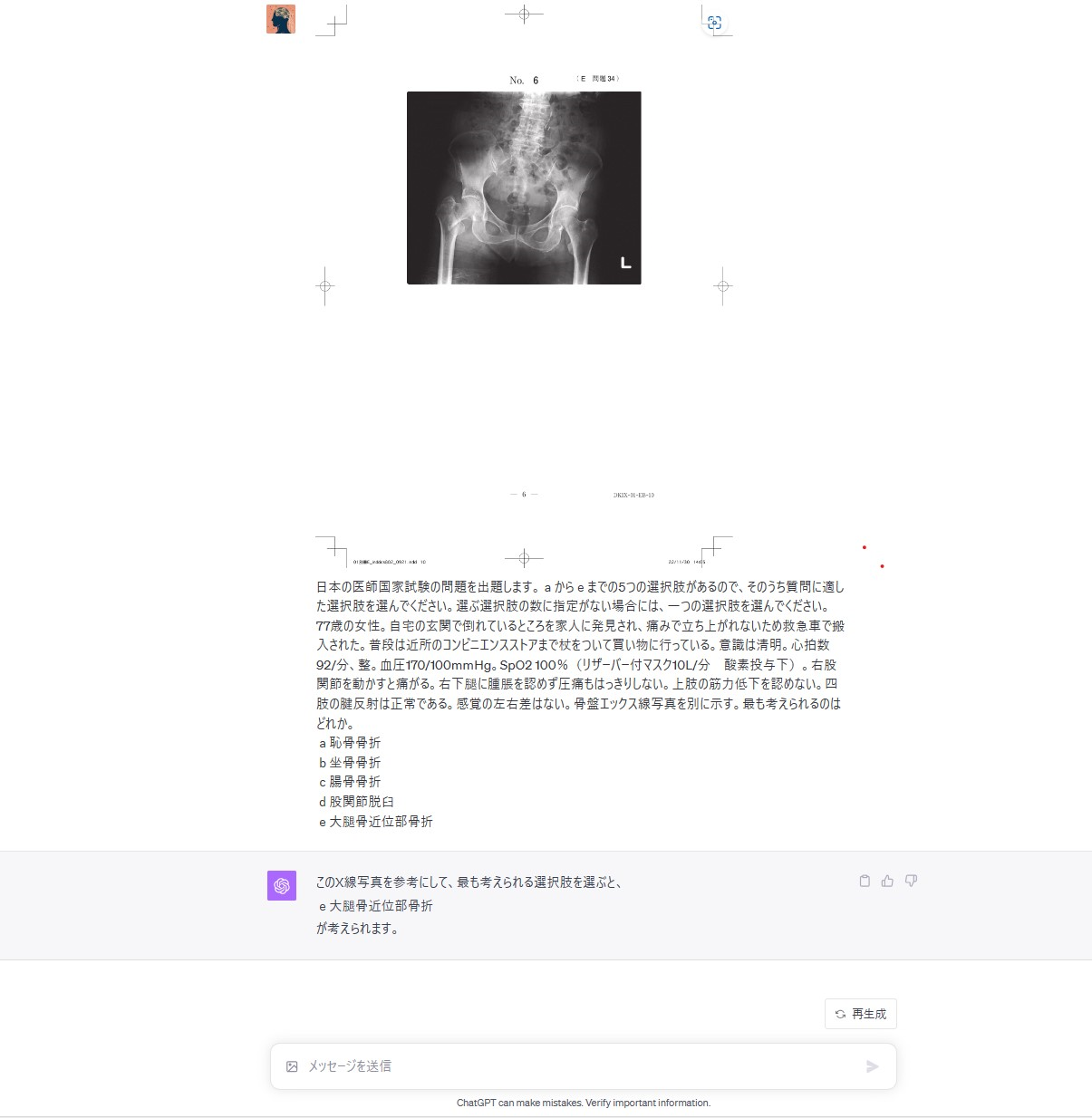


Figure S3. Screenshot of an actual ChatGPT-4V(ion) response: question with pathological image


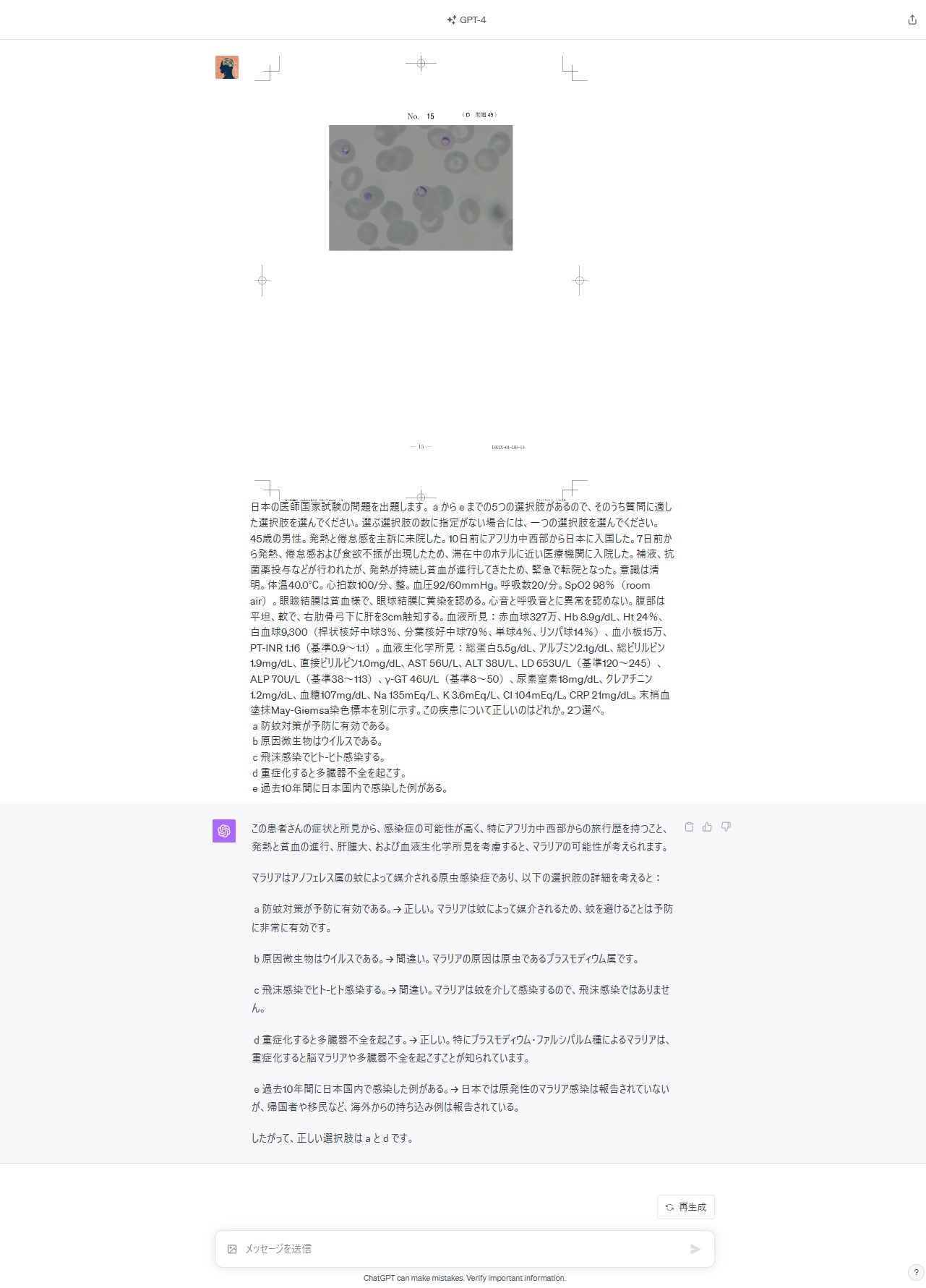


Figure S4. Screenshot of an actual ChatGPT-4V(ion) response: question with table


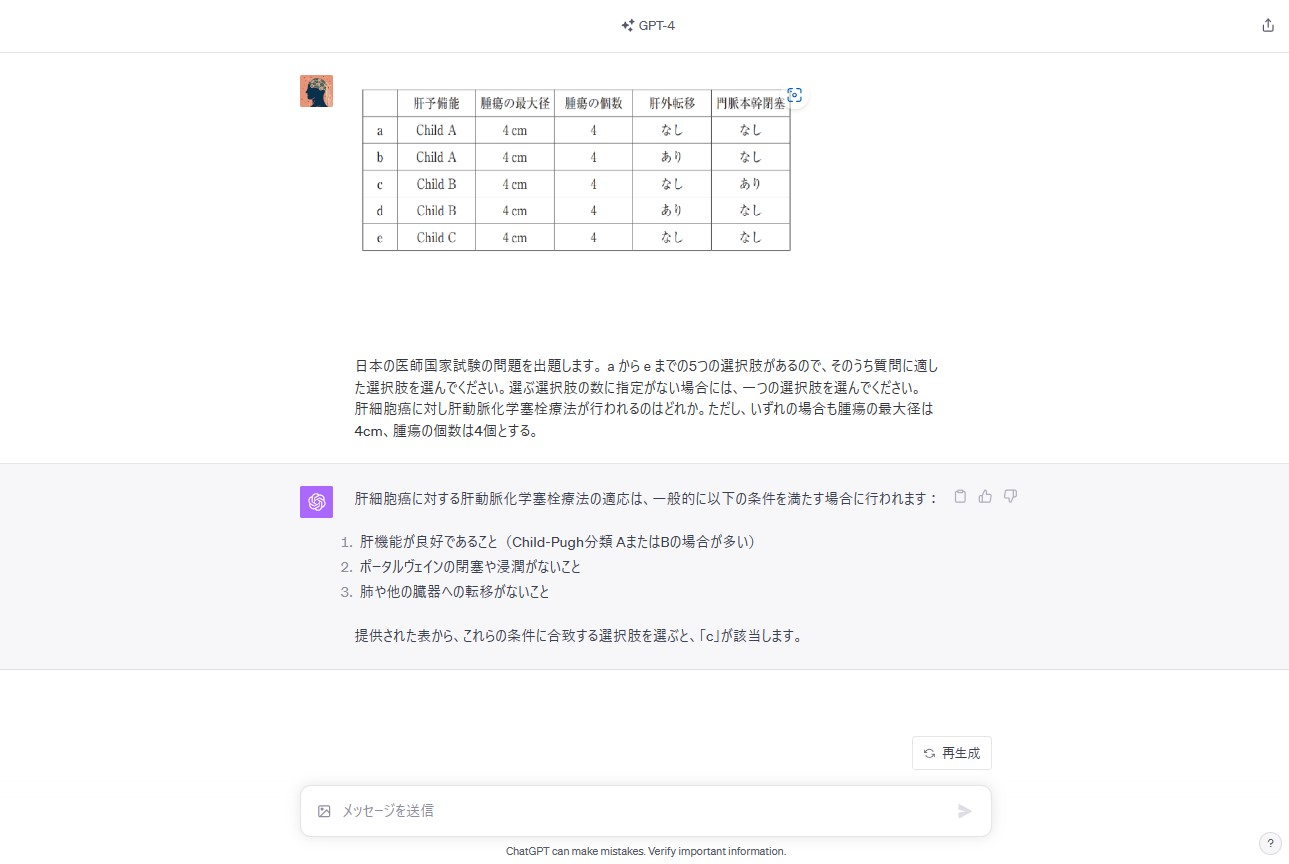


Figure S5. Screenshot of an actual ChatGPT-4V(ion) response: question with table


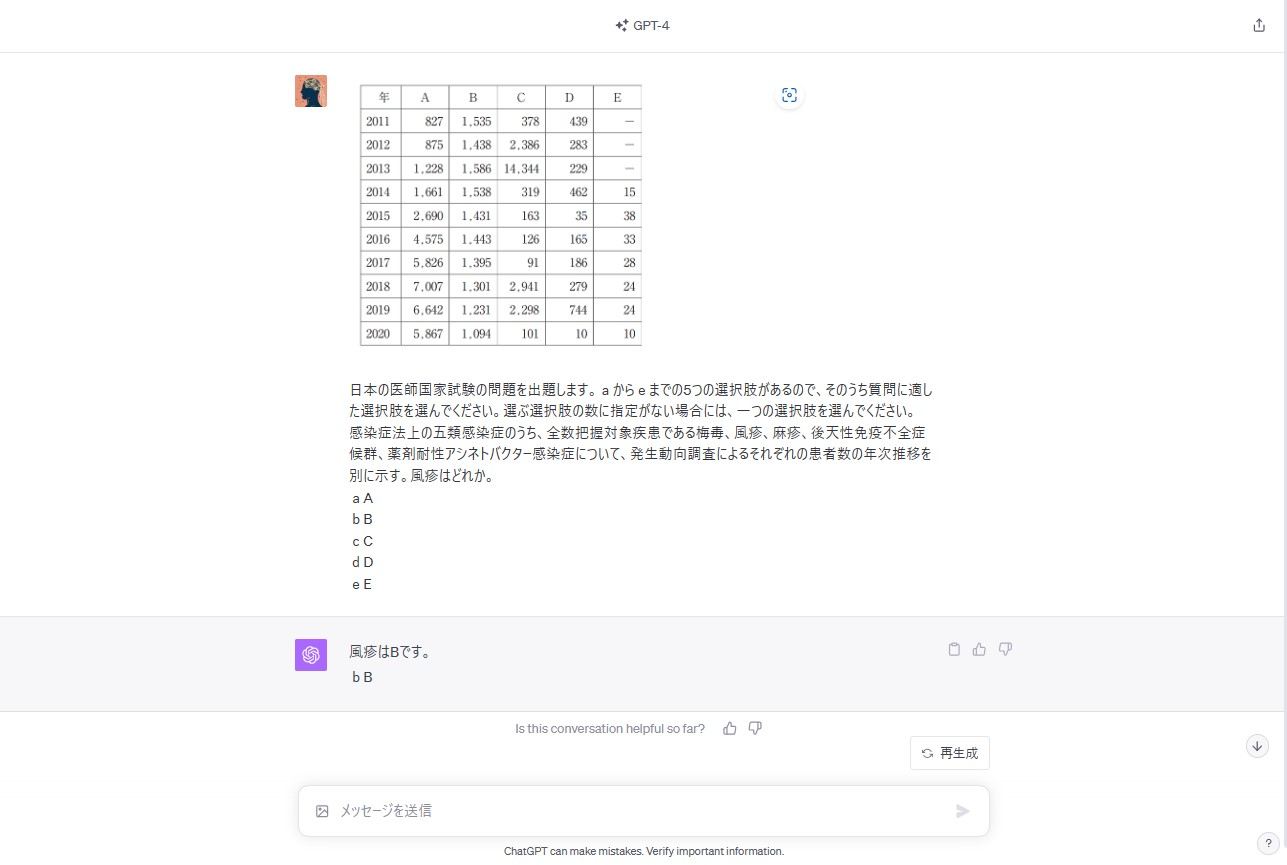

Supplement: Multimedia Appendix 1 [file mededu-v10-e54283-s001.docx]
