# Supplementary material for: The Performance of ChatGPT-4V in Interpreting Images and Tables in the Japanese Medical Licensing Exam
Source: JMIR Med Educ. 2024 May 23;10:e54283. doi: 10.2196/54283 (PMC11148840; doi:10.2196/54283)
Supplement: Multimedia Appendix 2 [file mededu-v10-e54283-s002.docx]

Supplementary File 2

For this study, ChatGPT Plus (default settings), based on the GPT-4V model, was used without any customized instructions, web search functionalities, or extension plugins. The 117th JMLE, held on February 4 and 5, 2023, questions and examinee data were also used in the study.

This exam includes 400 questions segmented into three categories: essential knowledge (assessing both the critical medical knowledge and ethics that a physician should possess), general clinical inquiries (regarding various diseases), and targeted disease questions (evaluating the knowledge of individual diseases) [1]. These questions were subdivided into three categories: general questions gauging the comprehension of a particular subject; clinical queries necessitating case depiction and clinical judgment; and clinical sentence queries comprising multiple questions within one case scenario. To pass the 117th JMLE, candidates must achieve at least 80% on the essential knowledge segment and 74.6% on the other questions [1].

Questions that the Ministry of Health, Labour, and Welfare explicitly stated would be excluded from the scoring (n=5) and those containing underlined segments (n=9) that ChatGPT cannot process were excluded; therefore, 386 questions were included in the final analysis.

The JMLE questions and their associated multiple-choice options were presented in native Japanese, consistent with official examination guidelines. The instructions for operating ChatGPT were also rendered in Japanese. The standard guidelines are as follows:

*“You will be presented with questions from the Japanese National Medical Examination. Options ranging from a to e will be provided, and you are to select the most fitting answer. Unless specified, restrict your choice to a single option.”*

The determination of "correct" responses when posing questions to ChatGPT was grounded in the JMLE's answers available on the MHLW's website [1]. Responses were deemed "correct” if they were unambiguously accurate and if they adhered to the directives in the content of the question. Ambivalent responses, blatant errors, and answers suggesting an undue number of choices were considered incorrect.

5. Ministry of Health, Labour and Welfare. *Announcement of Successful Passage of the 117th National Medical Examination* (Japanese). 2023. <https://www.mhlw.go.jp/general/sikaku/successlist/2023/siken01/about.html>. [26 Oct 2023]
